# A Novel Smartphone-Based Intervention Aimed at Increasing Future Orientation via the Future Self: a Pilot Randomized Controlled Trial of a Prototype Application

**DOI:** 10.1007/s11121-023-01609-y

**Published:** 2023-11-17

**Authors:** Esther C. A. Mertens, Aniek M. Siezenga, Job van der Schalk, Jean-Louis van Gelder

**Affiliations:** 1 Institute of Education and Child Studies, Leiden University, Leiden, Netherlands; 2 Department of Criminology, Max Planck Institute for the Study of Crime, Security and Law, Freiburg, Germany

**Keywords:** Future self-identification, Future orientation, Smartphone application intervention, mHealth, Randomized controlled trial (RCT), Goal achievement, Positive development, Self-defeating behavior

## Abstract

We developed and tested a smartphone-based intervention, FutureU, that aims to stimulate future-oriented thinking and behavior by strengthening the degree to which people identify with their future self. In order to examine the potential of this intervention prototype and opportunities for further optimization, we evaluated 1) the immediate and long-term efficacy of the intervention, and 2) intervention effects after each of three intervention modules. To this end, we conducted a randomized controlled pilot study among first-year university students (*N* = 176). Results showed a decrease in goal commitment immediately after the intervention. At 3-months follow-up, trends showed an increase in future orientation and in self-efficacy. During the intervention, there was a positive effect on vividness of the future self after the first module. Although there is scope for improvement, the findings highlight the potential of the intervention to increase people’s future-oriented thinking and behavior.

**Supplementary Information:**

The online version contains supplementary material available at 10.1007/s11121-023-01609-y.

## Introduction

Shortsighted behavior, i.e., favoring immediate gains over potential future costs, can negatively impact people’s development in both the psychological and the social domain (Steinberg et al., 2009). The preference for immediate gratification often coincides with discounting the future, which generally results in negative outcomes, such as self-defeating behavior (e.g., delinquency, substance use, unhealthy lifestyle; e.g., Hershfield et al., 2009; Rutchick et al., 2018). In contrast, considering possible future consequences when making decisions has been associated with positive outcomes, such as enhanced self-esteem and goal-directed behavior (e.g., Schmid et al., 2011; Zimbardo & Boyd, 1999), and refraining from negative behavior (Carmi, 2013). Despite the positive effects future orientation can have on one’s development, people generally find it difficult to make future-oriented choices (Hershfield, 2018). Young people in particular are typically oriented towards the present, find it difficult to plan ahead, and struggle to envisage the longer term consequences of their decisions. This youthful shortsightedness has been suggested as an underlying cause of poor and risky decision making (Steinberg et al., 2009). Therefore, increasing future orientation may decrease negative behavior and foster positive development.

Existing interventions that address future orientation often aim to change specific behaviors, such as food purchases (Hollis-Hansen et al., 2020) or academic performance (Nurra & Oyserman, 2018). Although an intervention addressing one specific behavior can be helpful for people struggling with this exact problem, it misses the opportunity to explicitly cultivate future orientation and related outcomes in multiple domains, both negative (e.g., self-defeating behavior) and positive (e.g., self-esteem).

We developed a novel smartphone-based intervention, FutureU, that aims to stimulate future-oriented thinking and behavior more broadly. In the present pilot study, we tested the efficacy of the FutureU smartphone application (app) prototype among a relatively young population (i.e., university students). That is, we tested intervention effects after each of three intervention modules, immediately following the intervention, and at 3-months follow-up, with the intention of gaining insight into the potential of the intervention on a broad range of outcomes and opportunities for further development of the app.

### Future Self-Identification

Preferences in intertemporal decision making have been viewed as a function of discrepancies between the needs and wants of a ‘present self’ and those of a ‘future self’ (Frederick et al., 2002). People are only willing to make choices in which they prioritize the future over the present when they identify with their future self (Hershfield, 2018). Such future self-identification has been argued to depend on the vividness of the imagined future self, the feelings towards this self, and the degree of perceived similarity and connectedness (i.e., relatedness) with it (Bixter et al., 2020). A desire for immediate gratification and temporal discounting may be more pronounced when people lack a vivid image of the future self, feel negatively about their future self, and/or lack a sense of relatedness to this future self.

Research has related (aspects of) future self-identification, which is also referred to as future-self continuity (e.g., Hershfield, 2011), to behavior in various domains. For example, increases in vividness of the future self have been related to reductions in delinquency (Van Gelder et al., 2015) and other self-defeating behavior (Van Gelder et al., 2022). Rutchick et al. (2018) have shown that a stronger sense of relatedness (i.e., similarity and continuity) to the future self was associated with better subjective health and with increased exercising. In addition, a stronger sense of continuity of the future self has been shown to be related to increased financial savings (Hershfield et al., 2011).

The FutureU intervention is based on the future self-identification framework. During the intervention, participants are stimulated to actively contemplate their future self and discover who this self is. We theorize that by doing so, participants gain a clearer and more vivid image of the future and their future self, develop (stronger) positive feelings towards this future self, and feel more related to this self. Together, this is assumed to result in a stronger identification with the future self, which, in turn, increases future-oriented thinking and behavior.

### Mental Time Travel

To increase people’s motivation and drive to consider the future consequences of their behavior, prior interventions have implemented Episodic Future Thinking (EFT) – a form of mental time travel (Suddendorf & Corballis, 2007). EFT is an intervention technique in which people are asked to vividly imagine and describe a realistic positive event or experience that could happen in their personal future (Hollis-Hansen et al., 2020). This technique builds on humans’ ability to pre-live events by mentally projecting themselves to the future which allows them to foresee, shape, and plan specific future events (Suddendorf & Corballis, 2007). By simulating the experience of receiving a larger future reward by deferring an immediate smaller reward, more farsighted behavior can be motivated (Schacter et al., 2017). EFT-based interventions have shown positive effects on specific financial and health related intertemporal choices (Rösch et al., 2021), such as food purchases (Hollis-Hansen et al., 2020), impulsive eating (Daniel et al., 2013), smoking (Stein et al., 2016), and monetary discounting (Daniel et al., 2013; Stein et al., 2016). The general finding is that intervention effects become stronger the more vividly the prospective event is imagined, underscoring the important role of vividness in EFT (Daniel et al., 2013; Rösch et al., 2021). We integrated EFT in the FutureU intervention to bolster a vivid imagination of the future self and future achievements.

### Smartphone-Based Interventions

Implementing interventions via technology, such as smartphone apps, provides unique opportunities compared to more traditional (face-to-face) interventions. For example, intervention apps can be programmed to automatically deliver intervention content and facilitate large-scale implementation. One major advantage is the possibility of apps to expose participants to intervention content on a regular basis. For most people, their smartphone is integrated in daily routines enabling its use throughout the day wherever and whenever they want (Linardon et al., 2019; Schoeppe et al., 2016). To remind users to engage with the app, preprogrammed push notifications can be sent (Linardon et al., 2019). Multimedia tools can provide content in different formats, such as video, audio, text, and graphic illustrations (e.g., Tielman et al., 2017).

Smartphone-based interventions have been shown to be effective across a variety of domains, such as mental health (e.g., internalizing problems, stress, quality of life; Donker et al., 2022; Linardon et al., 2019) and lifestyle choices (e.g., diet, sedentary behavior, physical activity; Fanning et al., 2017; Schoeppe et al., 2016). These positive effects have been related to various intervention components of apps. In their meta-analysis on smartphone-based interventions for mental health problems, Linardon et al. (2019) showed that stronger effects were related to the inclusion of push notifications as engagement reminders, supportive messages, and personalized feedback. Fanning et al. (2017) in their randomized factorial trial and Schoeppe et al. (2016) in their systematic review also showed a positive effect of (performance) feedback on intervention effects and found indications that an integrated goal-setting component was related to stronger intervention effects. These findings suggest that these specific components, i.e., push notifications, supportive messages, feedback, and goal-setting, may be effective ingredients of smartphone-based interventions.

The FutureU app contains several components that have been related to positive intervention effects in previous studies. More specifically, the FutureU app is designed for daily use and sends push notifications to remind users to interact with the app. The app contains a tool to work towards personal goals (i.e., goal-setting component), sends personalized messages (e.g., referencing participants’ personal goals), and provides supportive feedback (e.g., giving compliments and encouragement). Moreover, we capitalized on the multimedia possibilities provided by apps to expose participants to a three-dimensional visual rendering of their 10-year older future self. This visual presentation may stimulate the development of a more vivid image of the future self (McMichael et al., 2022), which, in turn, plays an important role in both future self-identification and EFT.

Although implementation of an intervention through technology, such as smartphone apps, has several advantages, it also comes with the challenge of translating theories of change (often examined in face-to-face interventions) into features of the technology (Fairburn & Patel, 2017). While many studies have focused on translating theories of change into web-based interventions, translation of theories into smartphone app features remains largely understudied (Fairburn & Patel, 2017). Therefore, we not only examined overall efficacy, but also intervention effects after each intervention module – each of the modules is based on different theories of change – to assess the degree to which the theories appeared successfully translated into features of the app. This knowledge can be used to inform the development of new intervention apps and identify opportunities for optimization of existing ones, in particular the next iteration of the FutureU app.

### Present Study

The aim of the present pilot study was to evaluate the potential of a prototype intervention app and identify opportunities for further optimization. To this end, we studied: 1) the immediate and long-term efficacy of the FutureU app prototype in stimulating future orientation, decreasing negative behaviors, and fostering positive development, and 2) intervention effects after each module, in order to get an indication of which modules successfully translated theories of change into technological features. First, we analyzed a broad range of primary (e.g., future self-identification, self-defeating behaviors) and secondary (e.g., psychosocial wellbeing, self-efficacy) outcomes. Second, we focused on a subsample of behavioral and cognitive outcomes that have the potential to change over the course of a week (i.e., future self-identification, self-defeating behavior, psychosocial wellbeing, weekly goal achievement). Intervention effects were analyzed in comparison to an active, goal-setting, control condition in order to examine effects of the app over and above the potential effects of setting goals (Van Lent & Souverijn, 2020).

The FutureU intervention was implemented among first-year university students. This population of relatively young people was deemed particularly suitable for our purposes as the transition from high school to university involves a transformational life event as well as a change of context. During such transitions, people are known to more often take a ‘big-picture’ view of their life, which makes it a suitable moment to intervene (Dai et al., 2014). We hypothesized that the intervention would increase future orientation and positive development, and decrease negative behaviors during, immediately after, and three months after the intervention.

## Method

### Design and Procedure

**Fig. 1:**
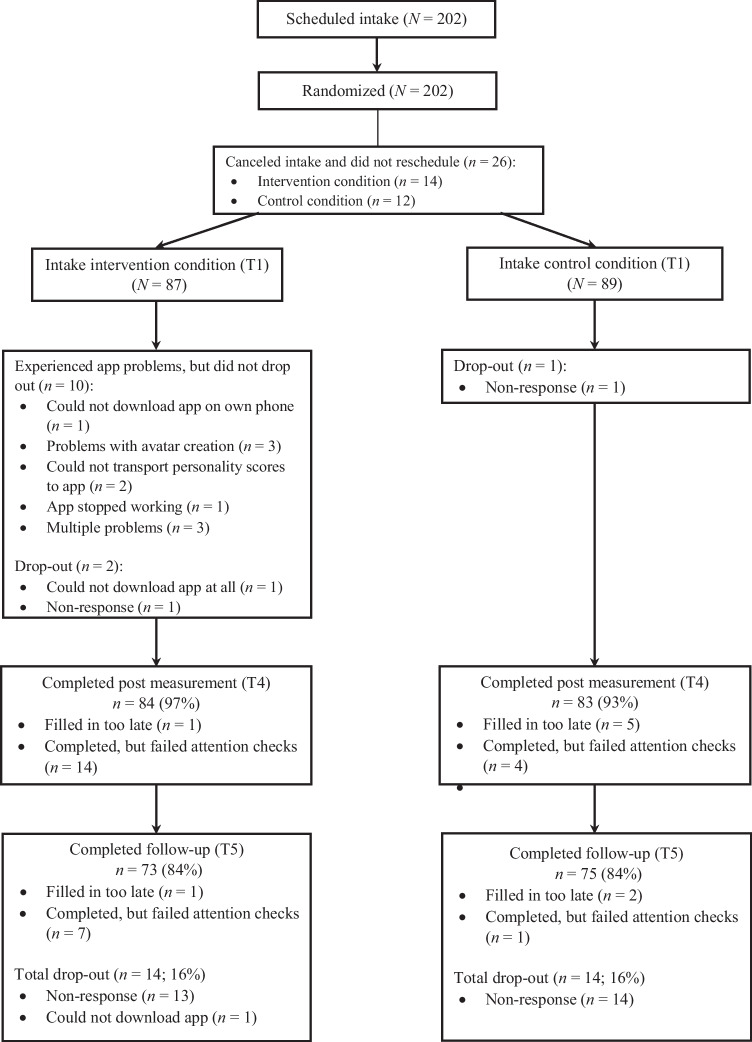
Flow chart

The intervention was examined in a Randomized Controlled Trial (RCT) with two conditions: 1) a smartphone-based intervention condition, and 2) an active control condition. Participants were randomly assigned to a condition using an online number generator and block randomization with blocks of six on a 1:1 ratio (conducted by AS), meaning that within a block three participants were allocated to each condition. See Fig. 1 for the flow chart.

Both conditions started out with an intake at the university’s lab during which participants gave active informed consent, completed the baseline questionnaire, and set two goals. Additionally, participants in the smartphone condition took a photo of their face (a ‘selfie’), which was digitally aged and turned into an avatar representing their future self (see Supplementary materials for details about the software used in this process). Subsequently, they installed the FutureU app on their smartphone.

Participants completed digital questionnaires at baseline (i.e., during intake; T1), immediately following each of the three week-long intervention modules (T2 and T3), immediately after the intervention (T4), and 3 months after the end of the intervention (T5). In the control condition parallel timepoints were used. Given that all participants completed the questionnaires multiple times, potential re-test effects would appear in both conditions and cannot account for differences between conditions. Participants received compensation for completing the questionnaires (course credits or money). Data was collected from October 2021 to June 2022. This trial was approved by the Ethics Board of the Institute of Education and Child Studies at Leiden University (ECWP2021-320) and registered in the Netherlands Trial Register number NL9671 (see Mertens et al., 2022 for the study protocol).

### Participants^1^

Participants were first-year university students (*N* = 176) enrolled at a university in the Netherlands. Most participants were enrolled in Pedagogical Sciences (57%) or Psychology (38%). Students were on average 19.64 years old (*SD* = 2.81) and mostly female (*n* = 155, 88%). There were no differences between conditions at baseline for age (Intervention *M*_*age*_ = 19.71; Control *M*_*age*_ = 19.21; *F*(1,174) = 1.39, *p* = .240, η_partial_^2^ = .008). Conditions did differ on gender distribution with slightly fewer female participants in the intervention condition than in the control condition (Intervention 82% female; Control condition 94% female; χ^2^(1) = 8.29, *p* = .004, φ = .218). Missing data (6.4% in total) could be regarded as missing at random (see Supplementary materials for details).

### FutureU Intervention Condition

Participants in the FutureU intervention condition started with an intake during which they set a goal for the coming month and a goal for the coming year (i.e., a monthly and a yearly goal). The goal-setting procedure was guided by the experimenter to ensure that goals followed the SMART-goals model and Zimmerman’s criteria (Ogbeiwi, 2021) resulting in specific, measurable, and challenging but attainable goals – goal characteristics, which are most likely to have a positive effect on performance (Van Lent & Souverijn, 2020). Subsequently, participants independently formulated a goal for the week, intended as a first step towards achieving their monthly goal, using the SMART criteria. At the end of Week 1 and Week 2 of the intervention, participants independently set a new goal for the following week.

**Fig. 2:**
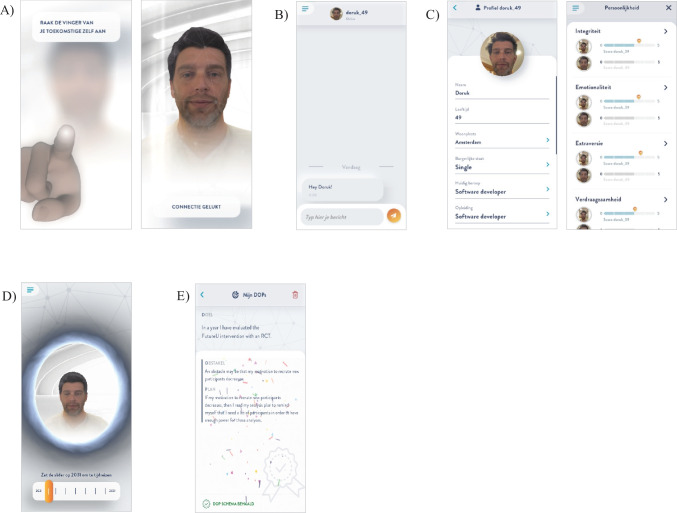
FutureU application screenshots of **A** Connection Mechanic, **B** Chat, **C** Personal Profile and Personality Menus, **D** Time Travel Portal, and **E** MCII Goal Schema

The general recurring features of the app are an interaction in which users connect with their future self (i.e., a ‘connection mechanic’), a (scripted) chat conversation with the future self, and push notifications. Participants access the app via the connection mechanic in which they touch their future self’s (virtual) finger. This action unblurs the screen and reveals an image of the future self based on the aged selfie (see Fig. 2A). In the chat (see Fig. 2B), participants ‘interact’ with their future self, receive psychoeducation, answer questions about their future, and receive instructions for the assignment of that day. Additionally, to keep the chat interaction engaging, the future self occasionally makes jokes, uses emojis, and sends funny pictures. The chat is scripted in such a way that interaction is suggested, for example, by directly addressing the participants and asking them multiple choice and open questions (e.g., “Do you already have an idea about what you can do in the coming days to achieve your weekly goal?”). The chat is scripted in such a way that participant responses are inconsequential for the flow of the chat so that all participants receive identical intervention content. Furthermore, each day of the intervention participants receive a push notification informing them that there is a chat message from their future self. In addition, every other day they receive a push notification with a general remark by their future self, (e.g., “I heard a good quote this morning: “Live for today, prepare for tomorrow!”. Totally us!”).

#### Intervention Modules

The app is divided into three consecutive, week-long, modules, each containing core features based on different and multiple theories of change (see Supplementary materials Table S1). The different features unlock as participants progress through the intervention.

**Module 1** The aim of the first module is twofold: 1) to increase vividness, familiarity, and identification with the future self, and 2) to stimulate thinking about one’s future circumstances and personality. To this end, participants fill in a personal profile of their future self, modeled on personal profile information common on social media. This profile contains information about demographics (e.g., place of residence, work experience) as well as information about skills, accomplishments, and life events. Hence, participants are encouraged to think about their future life circumstances in 10 years. Furthermore, participants receive psychoeducation about structural models of personality and personality change via a brief animated video clip embedded in the chat. In a personality overview screen, they are shown their own current personality scores (based on the personality questionnaire they completed at baseline) and norm scores. Subsequently, they can indicate what their desired score of their future self would be on these dimensions (see Fig. 2C).

**Module 2** The second module aims to stimulate future-oriented decision-making and changing attitudes and behavior in favor of the future self by practicing with temporally distanced perspective taking. Participants are presented with a short animated video providing psychoeducation about psychological and temporal distanced decision-making. They then practice temporally distanced perspective taking in a ‘time travel portal’. In this portal, they see their future self on screen, ostensibly at the other side of the portal. They first address their future self in third person to explain an issue they are facing in their daily life (this is recorded by the app). Subsequently, they ‘time travel’ and switch to the perspective of their future self, and now face their present self. They are presented with their own recording, and provide feedback. Participants switch perspectives multiple times during each session (see Fig. 2D).

**Module 3** This module focuses on growth mindsets (Dweck & Yeager, 2019) and how to set and achieve challenging goals. In two separate animated videos, participants learn about the purpose and nature of growth mindsets, and how Mental Contrasting and Implementation Intentions (MCII; Oettingen & Gollwitzer, 2010) can be applied when working towards goals. Participants practice with MCII by following its steps for their personal goals, which are saved in the app (see Fig. 2E).

#### Treatment Adherence

The FutureU app is developed for daily use for three consecutive weeks (i.e., 21 days) for approximately 5 min or less each day. Eight (9.20%) participants used the FutureU app every day during the intervention period and 32 (36.78%) participants almost every day (check-in range = 16 – 20 days). In contrast, 11 (12.64%) participants used the app only a few days (check-in range = 2 – 8 days). In total, 10 (11.49%) participants experienced technical problems with the app and 1 (1.15%) participant could not download the app. Most participants checked-in at least once during Module 1 (*n* = 86, 98.85%), during Module 2 (*n* = 82, 94.25%), and during Module 3 (*n* = 78, 89.66%). On average, participants used the app for 14 days (*SD* = 4.73; Median = 15 days) with check-in sessions lasting on average 4.39 min (*SD* = 1.71) per day. Generally, treatment adherence was moderate.

### Control Condition

During intake, participants in the control condition followed the same procedure as participants in the intervention condition – i.e., setting a goal for the month, a goal for the year, and a goal for that week. As in the intervention condition, participants independently formulated a new goal for the coming week at the end of each week. In contrast to the intervention condition, participants in the control condition did not receive further support while working towards their goals.

### Measurements

#### Primary Outcomes

**Future Self-Identification** The degree to which people identified with their future self was assessed with three subscales. All three subscales were assessed before, during, immediately after the intervention, and at 3-months follow-up.

**Vividness** Vividness measured the extent to which people had a clear and vivid image of their future self (based on Van Gelder et al., 2015). The scale consisted of 5 items (e.g., “I have a clear image of myself in 10 years.”) answered on a 7-point Likert scale (1 = *completely disagree* to 7 = *completely agree*; α = .92 – .95).

**Valence** Valence measured participants’ feelings towards the future self (based on Hershfield et al., 2009) and consisted of 1 item (“How do you feel when you think about your future?”). This item was answered on a 9-point scale ranging from negative to positive feelings with the Self-Assessment Manikin (Bradley & Lang, 1994).

**Relatedness** Relatedness measured the degree to which people felt connected to and similar to their future self. This was assessed with the 2-item Future Self-Continuity Measure (Hershfield et al., 2009). Both items were answered on a 7-point scale marked by pairs of circles that increase in overlap. The more circles overlap, the more connected or similar participants felt to their future self in 10 years (α = .57 – .83).

**Future Orientation** Future orientation was measured with the Future Orientation Scale (Steinberg et al., 2009), which assesses time perspective, anticipation of future consequences, and planning ahead. The questionnaire consists of 15 items with two pairs of opposite statements (e.g., “Some people spend very little time thinking about how things might be in the future, but other people spend a lot of time thinking about how things might be in the future.”). Participants were instructed to choose the statement that best described them and indicated whether that description was *completely true* or *a little bit true*. When the present-oriented description was chosen, a score of 1 (= *completely true*) or 2 (= *a little bit true*) was given. When the future-oriented description was chosen, a score of 3 (= *a little bit true*) or 4 (= *completely true*) was given (α = .84 – .85).

**Self-Defeating Behaviors** Behaviors with immediate gains though potential long-term costs were assessed with 16 items, based on Van Gelder et al. (2015), measuring self-defeating behaviors (e.g., “How often in the last week have you missed classes or work?”). Items were rated on a 5-point Likert type scale (1 = *never* to 5 = *more than 10 times*). Subsequently, responses were dichotomized into 0 (= *never*) and 1 (= *at least once*) for each item and summed to form one scale (α = .50 – .62). This concept was measured before, during, immediately after the intervention, and at 3-months follow-up.

**Goal Achievement** Goal achievement was measured with 3 items developed specifically for this study. Participants indicated to what extent they had thought about their goal, had worked towards their goal, and had achieved their goal, on a 5-point Likert scale (1 = *completely disagree* to 5 = *completely agree*). Goal achievement regarding the weekly goal was measured during and immediately after the intervention (Weekly goal achievement α = .67 – .82). Goal achievement regarding the monthly goal was measured immediately after the intervention and at 3-months follow-up (Monthly goal achievement T4 α = .76, T5 α = .80).

**Goal Commitment** Goal commitment was assessed with the Goal Commitment Questionnaire (Hollenbeck et al., 1989). This questionnaire consists of 7 items (e.g., “I think this goal is a good goal to shoot for.”) answered on a 7-point Likert scale (1 = *completely disagree* to 7 = *completely agree*; α = .60 – .86).

**Impulsiveness** Impulsiveness was measured with the Barratt Impulsiveness Scale short form, which assesses non-planning, motor impulsivity, and attentional impulsivity (Spinella, 2007). The questionnaire contains 15 items (e.g., “I do things without thinking.”) answered on a 4-point Likert scale (1 = *completely disagree* to 4 = *completely agree*; α = .85 – .86).

#### Secondary Outcomes

**Psychosocial Wellbeing.**Participants’ positive mental health was measured with the Warwick-Edinburgh Mental Wellbeing scale (Tennant et al., 2007) at baseline, immediately after the intervention, and at 3-months follow-up. This questionnaire consists of 14 items (e.g., “How often in the last week did you feel relaxed?”) answered on a 5-point Likert-type scale (1 = *never* to 5 = *always*; α = .87 – .91). For the interim measurements, the short version (7 items; Ng Fat et al., 2017) was used (T2 α = .79; T3 α = .81).

**Self-Efficacy** Self-efficacy, i.e., sense of competence to effectively deal with stressors of life, was assessed with the General Self-efficacy Questionnaire (Schwarzer & Jerusalem, 1995). The questionnaire consists of 10 items (e.g., “I can always manage to solve difficult problems if I try hard enough.”) answered on a 4-point Likert scale (1 = *completely disagree* to 4 = *completely agree*; α = .76 – .83).

**Self-Esteem** Global self-esteem was measured with the Rosenberg Self-esteem Scale (Rosenberg, 1965). The questionnaire contains 10 items (e.g., “On the whole, I am satisfied with myself.”) answered on a 4-point Likert scale (1 = *completely disagree* to 4 = *completely agree;* α = .85 – .88).

**Academic Achievement** The averaged academic result at the end of the first academic year was obtained from university records in August, i.e., the end of the academic year. In the Dutch education system scores range from 1 through 10 in which higher scores represent better results.

### Analyses

We used an intention-to-treat approach, implying that all participants who participated at baseline were included in the analyses regardless of whether they received the intervention or not. Missing data were estimated through Full Information Maximum Likelihood (FIML) procedures. The data were analyzed per outcome using autoregressive path models in R with the LAVAAN package (Rosseel, 2012). The outcome variable at each time point was regressed on the corresponding outcome variable of the previous time point. Additionally, the outcome variables at all time points were separately regressed on the dummy variable representing condition, with the control condition as reference group (including the outcome variable at baseline in order to control for initial difference between the conditions on the concerned outcome; see Fig. S1 Supplementary materials). Given that the main goal of the present study was to evaluate the potential of the FutureU app for establishing intervention effects and to identify opportunities to further develop the app, we highlight significant effects (*p* ≤ .050) as well as effects showing a trend towards significance (*p* ≤ .100).

For each time point and outcome variable, effect sizes were calculated as Cohen’s *d* with the baseline measurement as reference point (Cohen’s d = (*M*_change intervention_ / *SD*_pooled_) – (*M*_change control_ / *SD*_pooled_)). For variables without a baseline measurement (i.e., weekly goal achievement, monthly goal achievement, and academic results), effect sizes were calculated without baseline correction (Feingold, 2013). Effect sizes were calculated so that positive effect sizes indicated changes in the desired direction, that is, an increase for positive outcomes and a decrease for negative outcomes.

Effects established during or immediately after the intervention were analyzed in more detail, whenever possible (i.e., when interim measurements of the concerned outcome were available), by examining indirect effects via previous time points. This provided an indication of whether the effect after a certain module was mediated by the effect of an other module. Sensitivity analyses were conducted to test the robustness of the results (see Supplementary materials).

## Results

Descriptive statistics of the proximal and distal outcomes at baseline, during the intervention, immediately after the intervention, and at 3-months follow-up are presented per condition in Table 1.

**Table 1:** Descriptive statistics (M and SD) of the proximal and distal outcomes at baseline, during the intervention, immediately after the intervention, and at three months follow-up per condition

	Intervention condition					Control condition				
	T1	T2	T3	T4	T5	T1	T2	T3	T4	T5
	*M* (*SD*)	*M* (*SD*)	*M* (*SD*)	*M* (*SD*)	*M* (*SD*)	*M* (*SD*)	*M* (*SD*)	*M* (*SD*)	*M* (*SD*)	*M* (*SD*)
*Proximal outcomes*										
Future self-identification										
Vividness	3.24 (1.49)	3.83 (1.31)	3.95 (1.42)	3.83 (1.45)	3.68 (1.46)	3.50 (1.42)	3.77 (1.34)	3.82 (1.38)	3.77 (1.36)	3.64 (1.39)
Valence	6.59 (1.62)	6.49 (1.29)	6.39 (1.29)	6.45 (1.35)	6.37 (1.47)	6.73 (1.14)	6.61 (1.26)	6.51 (1.14)	6.59 (1.18)	6.62 (1.10)
Relatedness	3.76 (1.11)	3.83 (1.11)	4.05 (1.07)	4.16 (1.07)	4.11 (1.23)	3.94 (1.03)	3.95 (1.06)	4.08 (0.99)	4.20 (1.10)	4.13 (1.06)
Future orientation	2.95 (0.53)			2.86 (0.53)	2.98 (0.54)	3.11 (0.53)			3.08 (0.49)	3.09 (0.47)
Self-defeating behavior	4.89 (1.73)	4.39 (1.82)	4.31 (1.83)	3.77 (2.25)	4.78 (2.27)	4.16 (1.78)	3.81 (1.77)	3.65 (1.93)	3.52 (1.96)	4.53 (1.85)
Weekly goal achievement		3.42 (0.79)	3.23 (0.92)	3.47 (0.86)			3.39 (0.76)	3.36 (0.80)	3.38 (0.96)	
Monthly goal achievement				3.41 (0.83)	3.29 (0.91)				3.38 (0.96)	3.25 (0.99)
Yearly goal commitment	5.96 (0.54)			5.34 (0.88)	5.28 (1.17)	6.05 (0.58)			5.81 (0.74)	5.73 (0.84)
Impulsiveness	2.22 (0.40)			2.22 (0.35)	2.19 (0.41)	2.14 (0.43)			2.14 (0.44)	2.12 (0.40)
*Distal outcomes*										
Psychosocial wellbeing	3.57 (0.51)	3.53 (0.52)	3.43 (0.55)	3.56 (0.52)	3.46 (0.59)	3.55 (0.48)	3.59 (0.45)	3.54 (0.49)	3.60 (0.48)	3.61 (0.41)
Self-efficacy	2.93 (0.35)			2.97 (0.34)	2.99 (0.39)	2.85 (0.32)			2.88 (0.32)	2.87 (0.36)
Self-esteem	2.86 (0.46)			2.84 (0.47)	2.79 (0.51)	2.85 (0.41)			2.83 (0.45)	2.88 (0.44)
Academic achievement				7.26 (0.65)					7.18 (0.70)	

T1 = Baseline, T2 and T3 = Interim measurements, T4 = Post measurement, T5 = 3-Months follow-up

### Efficacy FutureU

Results from autoregressive path models are presented in Table 2. Immediately after the intervention, there was a large negative intervention effect (*d* = -.68) on yearly goal commitment. Participants in the intervention condition decreased more strongly on commitment to their yearly goal than participants in the control condition. At follow-up, there was no intervention effect, meaning that goal commitment of participants in the intervention condition did not decrease further after T4 compared to participants in the control condition. However, the difference between the two conditions compared to baseline remained relevant (*d* = -.64).

**Table 2:** Estimated regression coefficients of the outcomes per time point regressed on condition in the autoregressive path models and effect sizes per outcome and time point

	T1		T2		T3		T4		T5		Cohen’s *d*			
	*B*(*SE*)	*p*	*B*(*SE*)	*p*	*B*(*SE*)	*p*	*B*(*SE*)	*p*	*B*(*SE*)	*p*	T1-T2	T1-T3	T1-T4	T1-T5
*Proximal outcomes*														
Future self-identification														
Vividness	-.26(.22)	.242	**.27(.13)**	**.040**	.01(.11)	.931	-.01(.11)	.897	-.01(.16)	.965	**.22**	.27	.22	.21
Valence	-.14(.21)	.492	-.03(.14)	.834	-.07(.12)	.584	-.08(.15)	.590	-.08(.16)	.621	.01	.01	.00	-.08
Relatedness	-.19(.16)	.249	.04(.12)	.723	-.02(.10)	.816	.04(.10)	.687	-03(.14)	.817	.06	.14	.13	.15
Future orientation	**-.17(.08)**	**.036**					-.06(.04)	.164	***.07(.04)***	***.072***			-.11	***.09***
Self-defeating behavior	**.73(.26)**	**.006**	.11(.22)	.610	.30(.22)	.175	-.30(.25)	.227	.09(.28)	.746	.08	.04	.27	.27
Weekly goal achievement^1^			.02(.12)	.838	-.15(.12)	.211	.12(.13)	.373			.04	-.15	.10	
Monthly goal achievement^1^							-.05(.12)	.697	.07(.14)	.594			.04	.04
Yearly goal commitment	-.10(.08)	.255					**-.36(.11)**	**.001**	-.09(.14)	.523			**-.68**	-.64
Impulsiveness	.08(.06)	.199					.02(.03)	.633	-.02(.04)	.665			.00	-.02
*Distal outcomes*														
Psychosocial wellbeing	.01(.07)	.864	-.09(.05)	.102	-.06(.06)	.290	.01(.06)	.904	-.10(.07)	.155	-.16	-.27	-.12	-.35
Self-efficacy	.08(.05)	.101					.02(.04)	.659	***.09(.05)***	***.078***			.03	***.12***
Self-esteem	.01(.07)	.888					-.02(.04)	.570	-.06(.05)	.261			.00	-.23
Academic achievement^a^							.08(.10)	.464					.12	

Significant findings emphasized in bold, Trend towards significant findings emphasized in bold italics, T1 = Baseline, T2 and T3 = Interim measurements, T4 = Post measurement, T5 = 3-Months follow-up, Only a subsample of outcomes was assessed at interim measurements, Positive effect sizes indicate changes in the desired direction (i.e., an increase in positive outcomes and a decrease in negative outcomes)

^a^Variable had no baseline so Cohen’s *d* is calculated without baseline correction

At 3-months follow-up, there were trends towards significant positive intervention effects on future orientation and on self-efficacy. In both conditions, the observed scores of future orientation decreased immediately after the intervention. However, at follow-up, participants in the intervention condition showed a small increase in future orientation compared to baseline, whereas participants in the control condition still reported a small decrease on this outcome. The intervention effect (*d* = .09) was small based on the criteria of Cohen as well as based on the mean effect size distributions of universal interventions (Tanner-Smith et al., 2018). On self-efficacy, participants in the intervention condition seemed to improve slightly more than participants in the control condition. This effect (*d* = .12) would be interpreted as small based on Cohen’s criteria, though small to moderated based on the mean effect size distributions of universal interventions targeting self-concept outcomes (25^th^ percentile *d* = .06 and 50th percentile *d* = .17; Tanner-Smith et al., 2018).

### Intervention Effects After Each Module

After the first module, a positive effect on vividness of the future self emerged. Participants in the intervention condition had a clearer and more vivid image of their future self than participants in the control condition. The effect size (*d* = .22) was small based on the criteria of Cohen, but moderate based on the mean effect size distribution of universal interventions targeting self-concept outcomes (50^th^ percentile *d* = .17; Tanner-Smith et al., 2018). No other effects emerged on the other outcomes during the intervention (see Table 2).

## Discussion

As the integration of technology, in particular smartphone apps, in interventions is growing, there is a need for evidence-based intervention apps and knowledge about effective translation of theories of change into technological features. The present study evaluated a prototype of the FutureU app. Our purpose was, firstly, to investigate the potential of this specific intervention app to generate intervention effects and, secondly, to examine whether the modules successfully translated theories of change into app features. Our results indicated both negative and positive intervention effects. More specifically, there was a large negative effect on goal commitment immediately after the intervention, which remained relevant at follow-up. Furthermore, there were small to moderate positive effects on vividness of the future self (after the first intervention module), future orientation (at follow-up), and self-efficacy (at follow-up). We conclude from these findings that the FutureU app has potential, but also that there is a need to 1) further develop and optimize it, and 2) examine its potential among other populations than university students.

The negative effect on goal commitment could potentially be explained by imagination of the future self as well as by the study design. When participants set their goal for the year, they may have fantasized about their desired future and did not consider their expectations of success, thus the feasibility of the goal, when setting it. During the intervention, they were asked to imagine their future when their goal is obtained and subsequently identify which obstacles currently stand in their way of obtaining this goal (i.e., MCII used in module 3 of the intervention), posing to themselves the question whether their desired future can be realized. For some participants this may have triggered the thought that their goal is not attainable, which may have reduced their motivation to pursue this goal and, as a consequence, they may have lost their commitment to it (Oettingen et al., 2005). Another explanation relates to the design of the study. Participants set a goal for the year at the start of the intervention and reported their commitment to this goal over a period of time. During the intervention participants contemplated their future and who they want to be(come). This contemplation may have affected the way they think about their future self and clarified their vision of the future. Given that the way people think about and identify with their future self can affect their personal goals (Peetz & Wilson, 2008), participants’ goals may have changed during the intervention, resulting in reduced commitment to their previously set goals. To test this hypothesis, future research could focus on potential changes in long-term goals during and after an intervention aimed at stimulating future orientation.

The (trend towards) positive effects on vividness of the future self, future orientation, and self-efficacy signal the potential of the FutureU app. In particular, the effects on vividness and future orientation are promising as these are key outcomes of the intervention. Vividness plays an important role in both future self-identification and EFT (Rösch et al., 2021) and was hypothesized to play a pivotal role in establishing intervention effects. The increase in future orientation, albeit modest, implies that, after interacting with (a representation of) the future self, participants became more willing to give up something in the present to obtain benefits in the future, thought more about future consequences of their actions, and made more plans for the future (Steinberg et al., 2009) – characteristics of future-oriented thinking.

Taken together, our findings seem to support the theoretical framework of the FutureU intervention: Creating a vivid and clear vision of the future self can increase people’s propensity to favor the needs and wants of the future self over those of the present self. In the same line of reasoning, as people’s identification with their future self strengthens, they may also see the future self as a role model who successfully overcame obstacles in life, which, in turn, increases feelings of competence to effectively deal with stressors, i.e., self-efficacy (Scholz et al., 2002). The next step for future research is to test these potential mechanisms by analyzing whether vividness of the future self and future self-identification function as mediators in the intervention for cultivating future orientation and self-efficacy.

The effect on vividness of the future self emerged after the first intervention module, which tentatively suggests that the theories of change used in this module were successfully translated into technological features. Perhaps the intervention can be optimized by incorporating features of the first module into the other two modules or by referring back to the first module’s features in the other modules. The first module was based on theories about exposure to the future self (McMichael et al., 2022) and personality change over time (Thielmann & De Vries, 2021; Yeager, 2017). We translated these theories into app features by creating an avatar of the future self, presenting an animated video clip with psychoeducation about personality, and having participants fill out both a personal profile and a personality profile of their future self. It remains to be determined whether the effect on vividness was established by all these features in conjunction or only by a subset of these features. For future research, it would be interesting to test the effectiveness of the app features used in the first module separately in order to unravel which features were (particularly) effective. This could also further inform intervention theory, translation of theory into technology, and the development of other intervention apps.

### Strengths and Limitations

The findings of the present study should be interpreted in light of several strengths and limitations. First, prior to conducting this pilot RCT, the app had been thoroughly user-tested and iterated on the basis of informal small-scale pilot studies (see Mertens et al., 2022). Other strengths are the relatively large sample size and multiple measurement points allowing us to evaluate the intervention effects in detail.

A limitation of the study is that the sample consisted solely of university students who were mainly female. Although we specifically targeted this population of relatively young people, it limits the generalizability of our findings to other populations. Additionally, students received compensation for their participation in the study. Even though the compensation regarded completion of the questionnaires and not engagement with the intervention or for achieving goals, this may nevertheless have affected our results. Furthermore, as each intervention module was based on multiple theories of change that were translated into multiple technological features, we were unable to unravel the specific feature or (combination of) features that established the intervention effects after a module. In addition, not all outcomes could be assessed during the intervention, since we focused on outcomes that could reasonably change within a week. This limited our ability to analyze and explain the negative intervention effect on goal commitment after the intervention. It would be interesting for future research to examine whether this effect was related to specific intervention modules or whether it may have had an other explanation such as changed goals.

## Conclusion

In conclusion, the FutureU app prototype carries promise for stimulating young people’s future orientation, though further iterations are necessary to boost intervention effects. The positive intervention effects were in line with the future self-framework on which the intervention is based. The next step is to further develop the FutureU app, examine its working mechanisms, and test it among different populations. Furthermore, our findings suggest that our attempt to translate theories of exposure to the future self and personality change over time into technological features was successful as vividness of the future self increased after the module based on these theories. Our study constitutes a first step into studying how theories of change addressing vividness of the future self can be translated into smartphone app features. This knowledge can be used for the development of smartphone-based interventions focusing on a broad range of outcomes, as future self-identification has been shown to be relevant for various domains, including delinquency, lifestyle, and savings.

### Supplementary Information

Below is the link to the electronic supplementary material.

Supplementary file1 (DOCX 50 KB)

## Data Availability

Data that support the findings of this study are openly available in the Center for Open Science Online Supporting Information at https://osf.io/9jbrp/

## Footnotes

1 There was a COVID-19 related lockdown during data collection. To examine the potential effect of this lockdown, participants included before (*n* = 119) and after (*n* = 57) the lockdown were compared at baseline. We found no significant differences on either demographic or on the outcome variables, except for relatedness towards the future self (*F*(1,174) = 4.14, *p* = .043, η^2^_partial_ = .023). Participants included after the lockdown reported higher levels of relatedness at baseline than participants included before the lockdown. As only one significant difference with low relevance was found, we did not control for the lockdown in the analyses.

## Declarations

### Clinical Trial Registration

The trial is registered in the Netherlands Trial Register (number: NL9671) on 16 August 2021.
