# Supplementary material for: A Novel Smartphone-Based Intervention Aimed at Increasing Future Orientation via the Future Self: a Pilot Randomized Controlled Trial of a Prototype Application
Source: Prev Sci. 2023 Nov 17;25(2):392–405. doi: 10.1007/s11121-023-01609-y (PMC10891224; doi:10.1007/s11121-023-01609-y)
Supplement: Supplementary file 1 — Supplementary file1 (DOCX 50 KB) [file 11121_2023_1609_MOESM1_ESM.docx]

**Supplementary materials**

A Novel Smartphone-Based Intervention Aimed at Increasing Future Orientation via the Future Self: A Pilot Randomized Controlled Trial of a Prototype Application

Esther C. A. Mertens^1^, Aniek M. Siezenga^1,2^, Job van der Schalk^1^, & Jean-Louis van Gelder^1,2^

^1^ Institute of Education and Child Studies, Leiden University, the Netherlands

^2^ Department of Criminology, Max Planck Institute for the Study of Crime, Security and Law, Germany

Corresponding author:

Prof. dr. Jean-Louis van Gelder

[j.vangelder@csl.mpg.de](mailto:j.vangelder@csl.mpg.de)

Contents

[**Missing Data 2**](#_Toc129767149)

[**Avatar Creation 2**](#_Toc129767150)

[**Table S1 3**](#_Toc129767151)

[**Figure S1 4**](#_Toc129767152)

[**Sensitivity Analyses 5**](#_Toc129767153)

[**Analysis Plan 5**](#_Toc129767154)

[**Results 5**](#_Toc129767155)

[**Table S2 7**](#_Toc129767156)

[**Table S3 8**](#_Toc129767157)

[**References 9**](#_Toc129767158)

# **Missing Data**

Data were regarded missing when a participant did not complete a questionnaire at all, or in time (i.e., not within four days for the weekly assessments [T2 and T3], within eight days for the post assessment [T4], and within 16 days for the follow-up [T5]). Four, five, six, and three participants did not complete the questionnaires in time at T2, T3, T4, and T5 respectively. In total, 6.4% of the data were missing (Missing: T1 = 0.0%, T2 = 4.5%, T3 = 3.4%, T4 = 5.8%, T5 = 17.1%). Little’s MCAR test indicated that data were not missing completely at random (χ^2^ (558) = 642.48, *p* = .008).

Attrition analyses examining differences at each time point between participants who dropped out and who completed the study showed no significant differences on gender (χ^2^(1) = 1.16, *p* = .281, φ = .081), age or the outcome variables – based on α ≤ .01 due to multiple testing – (T1: *F*(11,164) = 1.18, *p* = .304, η^2­^_partial_ = .073; T2: *F*(6,161) = 2.41, *p* = .029, η^2­^_partial_ = .082; T3: *F*(6,163) = 2.09, *p* = .057, η^2­^_partial_ = .071; T4: *F*(12,151) = 1.54, *p* = .116, η^2­^_partial_ = .109). Given the lack of significant differences between drop-outs and completers, and that Little’s MCAR test tends to be conservative when applied to a large set of variables, we regarded the missing data as missing at random (Van Ness et al., 2007).

# **Avatar Creation**

Avatars representing participants’ future self were created using a combination of plug-in services and software specifically developed for the present research project by Orb Amsterdam ([www.orbamsterdam.com](http://www.orbamsterdam.com)). First, the photo of the participant’s face was age-progressed approximately 10 years using a custom built server and the online service of Change My Face ([www.changemyface.com](http://www.changemyface.com)). Second, the photo and aged-progressed image were converted into 3D avatars using software developed by Avatar SDK, version ‘Head 2.0’ ([www.avatarsdk.com](http://www.avatarsdk.com)) to create avatars representing the present self and the future self respectively.

# **Table S1**

*Modules of the Intervention* (Reprinted from the protocol paper of the current study Mertens et al., 2022, p. 4)

| Week | Module | Aim | Theory | Core features |
| --- | --- | --- | --- | --- |
| 1 | Future self and personality | Stimulating vividness, familiarity and identification with the future self and learn about their own and their future selves’ personality | - - - - Exposure to and vividness of the future self increases future orientation (McMichael et al., 2022).       - Incremental personality theory: The belief that personality can change over time can reduce problematic behaviors (Yeager, 2017).       - People’s willingness to change on personality traits in socially desirable ways increases after feedback on their current trait levels (Thielmann & De Vries, 2021). | - - - - Complete personal profile of the future self (e.g., work experience, skills, accomplishments)       - Current scores on personality traits with an indication of norm scores       - Psychoeducation that personality can change over time       - Set scores of personality traits of future self |
| 2 | Future self perspective | Practice with distanced perspective taking on problems to make future-oriented choices and increase self-insight with the potential to adjust attitudes and behaviors in favor of the future self | - - - - People make more future-oriented choices:  1. for others (i.e., Solomon’s paradox; Grossman & Kross, 2014) 2. when they have a vivid perception of the future self (McMichael et al.,2022) 3. when they can psychologically or temporally distance themselves from the situation (i.e., Construal level theory; Trope & Liberman, 2003).    - - - Wise reasoning is enhanced with third-person self-reflection (Grossmann et al., 2021) | - - - - Psychoeducation that people make more future-oriented choices when they distance themselves from the situation, and when they think about the long-term consequences       - Time travel portal to take future self perspective for giving compliment, advice, and motivation       - Participants address themselves in third-person       - Spoken interaction with playback of the recorded messages |
| 3 | Goal setting and achievement | Educate about a growth mindset to stimulate goal setting and practice Mental Contrasting and Implementation Intentions to foster goal achievement | - - - - Growth mindset: The belief that people’s abilities can develop over time. This mindset aids engagement in thoughts and behaviors to work towards goals (Dweck & Yeager, 2019).       - Mental Contrasting and Implementation Intentions (Oettingen & Gollwitzer, 2010): Method in which the desired future is contrasted with the current reality and then reflected upon obstacles in the way of attaining the desired future. Subsequently, a plan is formulated to implement behaviors to overcome obstacles, i.e., implementation intentions, in the format: If situation X, then I will do Y. | - - - - Psychoeducation that abilities can develop over time       - Video-clip explaining Mental Contrasting and Implementation Intentions       - Practice with Mental Contrasting and Implementation Intentions to work towards goals via filling in a scheme |

# **Figure S1**

*Diagram of Autoregressive Path Models, Including Indirect Paths, to Examine Intervention Effects*

Outcome T1

Outcome T3

Outcome T2

Outcome T4

Outcome T5

Condition

*Note.* Only a selection of the outcomes is measured at T2 and T3. Dotted grey arrows indicate modelled indirect effects.

# **Sensitivity Analyses**

## **Analysis Plan**

The robustness of the results was examined in two types of sensitivity analyses. For the first set of sensitivity analyses, we reran the autoregressive path models described above but excluded participants who 1) experienced problems with the app, and/or 2) failed at least one attention check at T4 and/or at T5^[[1]](#footnote-1)^. For the second set of sensitivity analyses, we reran the autoregressive path models and added gender as a covariate, as the conditions differed on gender distribution. If the covariate significantly explained variance on at least one of the time points in the model, we examined to what extent this affected the results. When interpreting the results of the sensitivity analyses, we particularly focused on the extent to which parameters and associations between condition and outcomes showed the same pattern as in the main analyses, rather than focusing solely on p-values as these are dependent on changes in sample size and standardized errors.

## **Results**

The first set of sensitivity analyses, in which participants who experienced technical problems and/or failed attention checks (*n* = 32) were excluded, showed results similar to the main analyses (see Table S2). Parameters of the intervention effects in the full sample (main analyses) were comparable to the parameters in the sensitivity sample (T2 vividness: *B*(*SE*)_main_ = .27(.13) vs. *B*(*SE*)_sens_ = .27(.15); T4 goal commitment: *B*(*SE*)_main_ = -.36(.11) vs. *B*(*SE*)_sens_ = -.34(.12); T5 future orientation: *B*(*SE*)_main_ = .07(.04) vs. *B*(*SE*)_sens_ = .06(.05); T5 self-efficacy: *B*(*SE*)_main_ = .09(.05) vs. *B*(*SE*)_sens_ = .09(.06)). The p-values slightly increased, in particular regarding future orientation, which could be due to the smaller sample size and larger standardized errors. Furthermore, it is noteworthy that in the sensitivity analysis a negative intervention effect at T2 was found on psychosocial wellbeing. While the parameters are comparable (*B*(*SE*)_main_ = -.09(.05) vs. *B*(*SE*)_sens_ = -.10(.05)), this relation was only significant in the sensitivity sample (*p*_main_ = .102 vs. *p*_sens_ = .048).

The second set of sensitivity analyses in which gender was added as a covariate to the model when it significantly predicted the outcome at at least one time point were also in line with the results of the main analyses (see Table S3). Gender was a significant covariate in the models of future orientation, self-defeating behavior, monthly goal achievement, yearly goal commitment, and self-efficacy. The parameters of the models with and without gender as covariate were roughly the same (T4 goal commitment: *B*(*SE*)_main_ = -.36(.11) vs. *B*(*SE*)_sens_ = -.30(.12); T5 future orientation: *B*(*SE*)_main_ = .07(.04) vs. *B*(*SE*)_sens_ = .09(.04); T5 self-efficacy: *B*(*SE*)_main_ = .09(.05) vs. *B*(*SE*)_sens_ = .09(.05)). The p-values changed somewhat (the effect of future orientation at T5 changed from a trend to a significant effect), though showed the same pattern as in the main analyses.

# **Table S2**

*Estimated Regression Coefficients per Outcome and Time Point for the Sensitivity Sample Excluding Participants Experiencing Problems or Failed Attention Checks (N = 144)*

|  | | |  | T1 | | T2 | | | T3 | | T4 | | T5 | |
| --- | --- | --- | --- | --- | --- | --- | --- | --- | --- | --- | --- | --- | --- | --- |
|  | | |  | *B*(*SE*) | *p* | *B*(*SE*) | *p* | *B*(*SE*) | | *p* | *B*(*SE*) | *p* | *B*(*SE*) | *p* |
| *Proximal outcomes* | | | |  |  |  |  |  | |  |  |  |  |  |
|  | Future self-identification | | |  |  |  |  |  | |  |  |  |  |  |
|  |  | Vividness | | -.28(.24) | .245 | **.27(.15)** | **.072** | .02(.13) | | .898 | .03(.13) | .812 | -.15(.18) | .401 |
|  |  | Valence | | -.05(.24) | .826 | -.09(.16) | .577 | -.08(.14) | | .575 | .21(.14) | .126 | -.12(.16) | .461 |
|  |  | Relatedness | | -.14(.18) | .457 | .02(.13) | .909 | -.12(.11) | | .295 | .08(.11) | .428 | -.06(.16) | .710 |
|  | Future orientation | | | **-.20(.09)** | **.031** |  |  |  | |  | -.03(.05) | .563 | .06(.05) | .234 |
|  | Self-defeating behavior | | | **.63(.29)** | **.027** | .18(.24) | .452 | .28(.22) | | .202 | -.20(.27) | .442 | -.01(.29) | .967 |
|  | Weekly goal achievement | | |  |  | .04(.13) | .767 | -.17(.13) | | .199 | -.09(.15) | .538 |  |  |
|  | Monthly goal achievement | | |  |  |  |  |  | |  | -.21(.13) | .122 | .17(.14) | .231 |
|  | Yearly goal commitment | | | -.06(.09) | .502 |  |  |  | |  | **-.34(.12)** | **.006** | -.16(.17) | .351 |
|  | Impulsiveness | | | .07(.07) | .295 |  |  |  | |  | .01(.04) | .737 | -.03(.04) | .467 |
| *Distal outcomes* | | | |  |  |  |  |  | |  |  |  |  |  |
|  | Psychosocial wellbeing | | | .10(.08) | .217 | **-.10(.05)** | **.048** | -.07(.07) | | .280 | .04(.06) | .504 | -.12(.08) | .117 |
|  | Self-efficacy | | | .09(.06) | .110 |  |  |  | |  | .01(.04) | .826 | **.09(.06)** | **.096** |
|  | Self-esteem | | | .07(.08) | .385 |  |  |  | |  | -.02(.04) | .689 | -.03(.06) | .567 |
|  | Academic achievement | | |  |  |  |  |  | |  | .03(.11) | .781 |  |  |

*Note.* (Trend) significant findings emphasized in bold; T2 and T3 = Interim measurements; T4 = Post measurement; T5 = 3-Months follow-up; Only a subsample of outcomes was assessed at interim measurements.

# **Table S3**

*Estimated Regression Coefficients per Outcome and Time Point for the Sensitivity Analyses Regarding the Models in which Gender as Covariate was a Significant Predictor of at Least One Time Point (N = 175)^1^*

|  | | |  | T1 | | T2 | | | T3 | | T4 | | T5 | |
| --- | --- | --- | --- | --- | --- | --- | --- | --- | --- | --- | --- | --- | --- | --- |
|  | | |  | *B*(*SE*) | *p* | *B*(*SE*) | *p* | *B*(*SE*) | | *p* | *B*(*SE*) | *p* | *B*(*SE*) | *p* |
| *Proximal outcomes* | | | |  |  |  |  |  | |  |  |  |  |  |
|  | Future self-identification | | |  |  |  |  |  | |  |  |  |  |  |
|  |  | *Vividness* | | *-.26(.22)* | *.245* | *.24(.14)* | *.074* | *.03(.11)* | | *.778* | *-.06(.12)* | *.635* | *-.01(.15)* | *.942* |
|  |  | *Valence* | | *-.17(.21)* | *.429* | *-.03(.16)* | *.829* | *-.06(.12)* | | *.626* | *-.03(.14)* | *.830* | *-.14(.16)* | *.385* |
|  |  | *Relatedness* | | *-.18(.17)* | *.270* | *.04(.11)* | *.760* | *-.02(.09)* | | *.816* | *.06(.10)* | *.580* | *.02(.13)* | *.860* |
|  | Future orientation | | | -.13(.08) | .113 |  |  |  | |  | -.06(.04) | .177 | **.09(.04)** | **.033** |
|  | Self-defeating behavior | | | **.63(.27)** | **.018** | .25(.22) | .265 | .19(.20) | | .343 | -.35(.25) | .162 | .07(.28) | .817 |
|  | *Weekly goal achievement* | | |  |  | *.05(.12)* | *.690* | *-.16(.12)* | | *.191* | *.15(.14)* | *.294* |  |  |
|  | Monthly goal achievement | | |  |  |  |  |  | |  | -.06(.13) | .662 | .14(.14) | .312 |
|  | Yearly goal commitment | | | -.10(.08) | .219 |  |  |  | |  | **-.30(.11)** | **.004** | -.15(.15) | .298 |
|  | *Impulsiveness* | | | *.06(.06)* | *.344* |  |  |  | |  | *.02(.03)* | *.568* | *-.02(.04)* | *.575* |
| *Distal outcomes* | | | |  |  |  |  |  | |  |  |  |  |  |
|  | *Psychosocial wellbeing* | | | *.01(.08)* | *.624* | *-.09(.05)* | *.083* | *-.07(.06)* | | *.255* | *.02(.06)* | *.255* | *.02(.06)* | *.718* |
|  | Self-efficacy | | | .06(.05) | .212 |  |  |  | |  | .02(.04) | .576 | **.09(.05)** | **.077** |
|  | *Self-esteem* | | | *.02(.07)* | *.732* |  |  |  | |  | *-.02(.04)* | *.608* | *-.05(.05)* | *.346* |
|  | *Academic achievement* | | |  |  |  |  |  | |  | *.08(.11)* | *.472* |  |  |

*Note.* Gray italics report the results of the models in which gender was included as a covariate but was no significant covariate at any time point; (Trend towards) significant findings emphasized in bold; T2 and T3 = Interim measurements; T4 = Post measurement; T5 = 3-Months follow-up; Only a subsample of outcomes was assessed at interim measurements; ^1^One participant actively did not identify as male or female (answered ‘4 = *no answer*’) and could therefore not be included in these models.

# **References**

Mertens, E.C.A., Van der Schalk, J., Siezenga, A.M., & Van Gelder, J.-L. (2022). Stimulating a future-oriented mindset and goal attainment through a smartphone-based intervention: Study protocol for a randomized controlled trial. *Internet Interventions, 27*, article 100509. <https://doi.org/10.1016/j.invent.2022.100509>

Van Ness, P. H., Murphy, T. E., Araujo, K. L. B., Pisani, M. A., & Allore, H. G. (2007). The use of missingness screens in clinical epidemiologic research has implications for regression modeling. *Journal of Clinical Epidemiology, 60*(12), 1239-1245. <https://doi.org/10.1016/j.clinepi.2007.03.006>

1. We embedded two attention checks items in the T4 and T5 questionnaires instructing the participant to select a specific response category. These items served as indicator of participants’ (in)attentiveness. [↑](#footnote-ref-1)
